# Beyond ALS: split-hand syndrome in immune-mediated motor neuropathies

**DOI:** 10.1007/s00415-026-14006-6

**Published:** 2026-07-17

**Authors:** Luisa Kreß, Mai Abuzant, Daniel Zeller, Claudia Sommer

**Affiliations:** Department of Neurology, University Hospital Würzburg, Josef-Schneider-Str. 11, 97080 Würzburg, Germany

**Keywords:** Split-hand syndrome, Multifocal motor neuropathy, Multifocal acquired demyelinating sensory and motor neuropathy, Amyotrophic lateral sclerosis

## Abstract

**Background and aims:**

Split-hand syndrome describes selective wasting and weakness of the abductor pollicis brevis (APB) or first dorsal interosseous (FDI) muscles with relative preservation of the abductor digiti minimi (ADM). Beyond clinical definition, two neurophysiological ratios and one index have been proposed to quantify this pattern. It is considered a potential diagnostic criterion for amyotrophic lateral sclerosis (ALS). Its occurrence in immune-mediated neuropathies, as differential diagnoses, remains unclear. We aimed to investigate clinical and electrophysiological manifestations of split-hand syndrome in multifocal motor neuropathy (MMN) and multifocal acquired demyelinating sensory and motor neuropathy (MADSAM) compared to ALS.

**Methods:**

We prospectively examined 26 MMN, 16 MADSAM, and 22 ALS patients. All underwent neurological examination and neurophysiological measurements of compound muscle action potentials (CMAP) from the APB, FDI, and ADM bilaterally after median (APB) and ulnar nerve (FDI, ADM) stimulation. Split-hand ratios (APB/ADM; FDI/ADM) and split-hand index (SI) were calculated.

**Results:**

Clinical split-hand syndrome was present in 16/26 (62%) MMN, 7/16 (44%) MADSAM, and 12/22 (54%) ALS patients. Electrophysiological criteria (abnormal split-hand ratios or SI; ≥ 1 parameter fulfilled) were similarly frequent across groups (MMN 17/26, 65%, MADSAM 11/16, 69%, and ALS 16/22, 73%). CMAP ratios and SI did not differ between groups in the overall analysis and showed no correlation with disease duration or severity. Diagnostic models showed limited discriminatory power (area under the curve ≤ 0.61).

**Interpretation:**

Split-hand syndrome occurs in MMN, MADSAM, and ALS at comparable frequencies and lacks robust or consistent diagnostic discrimination across disease groups.

**Supplementary Information:**

The online version contains supplementary material available at 10.1007/s00415-026-14006-6.

## Introduction

The split-hand syndrome is a distinct pattern of dissociated muscle atrophy in the hands, characterized by predominant wasting of the abductor pollicis brevis (APB) as a thenar muscle and/or the first dorsal interosseous (FDI), with relative preservation of the abductor digiti minimi (ADM) as a muscle in the hypothenar eminence [1, 2]. First described by Wilbourn in 1994 [3], this selective muscle involvement has gained increasing recognition as a potentially useful diagnostic indicator for identifying patients with amyotrophic lateral sclerosis (ALS) [4, 5].

To improve diagnostic precision, various electrophysiological criteria have been proposed, with APB/ADM and FDI/ADM compound muscle action potential (CMAP) ratios, and the split-hand index (SI) being the most common used ones [6–9]. These criteria are frequently promoted to increase diagnostic sensitivity and specificity in ALS, with reported specificities ranging from 80% to 95% [10–12].

However, recent evidence has challenged the specificity of this phenomenon to ALS. Similar split-hand patterns have been described in other motor neuronopathies such as spinal (and bulbar) muscular atrophy [11, 13], Hirayama disease [14, 15], or in certain hereditary neuropathies like Charcot–Marie–Tooth disease [16]. These findings broaden the potential clinical spectrum of the split-hand syndrome, but systematic studies in treatable neuropathies remain lacking**.**

Among the differential diagnoses of ALS, especially in patients presenting predominantly with lower motor neuron signs, multifocal motor neuropathy (MMN) and multifocal acquired demyelinating sensory and motor neuropathy (MADSAM, also known as Lewis–Sumner syndrome) [17] are of particular relevance. While MADSAM often presents with sensory signs and symptoms, in contrast to MMN and ALS, these may be very subtle, particularly in the early stages and the disease may therefore mimic MMN or ALS [18]. MMN and MADSAM typically show abnormalities following peripheral nerve distributions, whereas ALS more often presents with a myotomal pattern consistent with motor neuron involvement; however, this distinction is not always clear-cut in clinical practice [19–21]. Despite their potentially similar initial clinical presentation, MMN and MADSAM differ considerably in treatment and prognosis [22]. Various diagnostic approaches, ranging from clinical signs and electrophysiological markers [22, 23] to imaging [24–26] and blood biomarkers [18, 27, 28], have been explored. Although isolated case reports have described split-hand patterns in immune-mediated neuropathies, no systematic study to date has evaluated its presence, diagnostic value, or distinguishing features in MMN or MADSAM.

This study addresses this gap by systematically evaluating the occurrence and diagnostic utility of the split-hand syndrome in treatable neuropathies, with a particular focus on MMN and MADSAM. Both clinical assessment and electrophysiological parameters (such as CMAP ratios and SI) are employed, and findings are compared to ALS data. Our aim is to determine whether distinctive split-hand patterns exist in MMN and MADSAM that may assist in differential diagnosis, improving early recognition, and preventing misdiagnosis or inappropriate therapy in patients with potentially treatable motor neuropathies.

## Methods

### Patient cohorts

A total of 26 (15 male) patients with MMN, 16 (12 male) patients with MADSAM, and 22 (15 male) patients with ALS were prospectively recruited between November 2021 and February 2025 at the Department of Neurology, University Hospital Würzburg. Enrollment took place during routine clinical visits. Diagnoses of MMN, MADSAM, or ALS were made prior to inclusion by a neurology specialist (CS, DZ) according to current diagnostic guidelines: EFNS/PNS criteria for MMN [29], EAN/PNS criteria for MADSAM [17], and the Gold coast criteria for ALS [30]. Exclusion criteria were comorbidities that hindered participation, severe alcohol or drug abuse, comorbid entrapment syndromes, and insufficient proficiency in German. An additional exclusion criterion for ALS was isolated bulbar manifestation. Medical reports were reviewed for comorbidities affecting peripheral nerve function. All participants underwent clinical examination and neurophysiological assessments. In addition to the prospective dataset, we retrospectively reviewed 22/26 (85%) MMN and 12/16 (75%) MADSAM patients at their first visit and at the time of initial diagnosis, provided they were treatment naïve. Four MMN and three MADSAM patients were excluded due to prior immunotherapy. From the prospective ALS cohort, we selected patients with a disease duration of < 2 years (18/22, 81%) to ensure comparability at the most critical diagnostic stage. The median time from symptom onset to diagnosis was 2 years (range 0–8) for MMN, 1 year (range 0–11) for MADSAM, reflecting retrospectively recalled initial symptoms; diagnostic evaluation occurred at the time of clinically manifest disease. This study was approved by the Ethics Committee of the University of Würzburg (#229/22). All participants provided written informed consent before enrollment.

### Clinical examination

All patients underwent a comprehensive neurological examination performed by experienced neurologists with a particular focus on assessing finger and hand muscle strength using the Medical Research Council (MRC) scale. The MRC scale ranges from five (normal strength) to zero (plegia), with muscle weakness defined as an MRC score of four or lower. Muscle atrophy was evaluated through visual assessment, categorizing atrophy as either present or absent for the respective muscles. Given the subjective nature of visual atrophy assessment, examinations were restricted to two experienced medical doctors (LK and CS) to ensure consistency. To quantify overall upper limb strength, the extended MRC sum score was calculated. This included the manual muscle strength determination of six muscle groups (shoulder abduction, elbow flexion, elbow extension, wrist extension, finger extension, finger spreading) bilaterally using MRC scale yielding a maximum total score of 60. In patients with neuropathy the overall disability sum score (ODSS) was used to assess the upper and lower limb functions (consisting of a checklist for interviewing patients and being scaled from zero = no limitations to five (upper limb) or seven (lower limb) = no purposeful movement) [31]. For patients with ALS, the Revised Amyotrophic Lateral Sclerosis Functional Rating Scale (ALSFRS-R) score [32] was determined. Split-hand syndrome was defined when at least one of the three criteria was met: clinical based on either MRC strength or visible atrophy pattern, or based on neurophysiological indices.

### Clinical split-hand syndrome criteria

The clinical criteria for split-hand syndrome were considered met under the following conditions: (i) preferential weakness of the APB and/or FDI, with relative preservation of the ADM, as determined by the MRC scale and defined as ∆MRC (ADM vs. FDI) ≥ 1 or ∆MRC (ADM vs. APB) ≥ 1; or (ii) visual inspection revealed muscle atrophy of the APB and/or FDI that was greater than that of the ADM, based on comparative assessment of muscle thickness.

### Neurophysiological studies and index calculations

Nerve conduction studies of the median and ulnar nerves were performed in all patients following standard procedures, electrode placement, and stimulation techniques [33]. Prior to measurement, extremity temperature was confirmed to be at least 32°C. After supramaximal nerve stimulation at the wrist the resulting CMAP amplitudes (peak to peak in [mV]) were recorded bilaterally over the APB (N. medianus), ADM, and FDI (N. ulnaris, each) by surface electrodes. For calculation of the split-hand indices, we used the (predominantly) affected side. In case of equally affected sides, we calculated the mean values between left and right.

The presence of the split-hand phenomenon was determined by using the criteria defined by *Wilbourn *et al*. 1994 *[3], which are met if

$$\frac{CMAP Amplitude APB}{CMAP Amplitude ADM}<0.6 and/or \frac{CMAP Amplitude FDI}{CMAP Amplitude ADM}<0.9$$

based on the SI, which is defined as follows [7]:

$$SI:\frac{CMAP Amplitude APB x CMAP Amplitude IOD1}{CMAP Amplitude ADM}<5.2.$$

### Statistical analysis

Statistical analyses were conducted using IBM SPSS Statistics version 29 (IBM Deutschland GmbH, Ehningen, Germany). Normality was assessed using the Shapiro–Wilk test. For normally distributed data, independent samples were compared using the t-test; otherwise, the Mann–Whitney U test was applied. The Kruskal–Wallis test was utilized for comparisons involving more than two non-normally distributed groups. Categorical variables were analyzed using Fisher’s exact test or the chi-squared (χ^2^) test. Spearman’s rank correlation was calculated to assess correlations. A p-value < 0.05 was considered statistically significant. Figures were generated with GraphPad Prism version 9.5.1 (GraphPad Software, San Diego, CA, USA).

To evaluate the diagnostic accuracy of split-hand-related criteria in differentiating ALS from MMN, two complementary analytical approaches were applied. First, three binary parameters, namely, clinical split-hand criterion (based on (i) MRC strength or (ii) atrophy pattern) and electrophysiological indices, were assessed individually via cross-tabulations. Their combined diagnostic performance was determined through binary logistic regression, with predicted probabilities subjected to receiver operating characteristic (ROC) curve analysis. In the second approach, three continuous electrophysiological parameters (CMAP ratios and the SI) were first evaluated separately through ROC curve analysis, then incorporated into a multivariate logistic regression model to assess their combined discriminative ability.

## Results

### Demographic data

Detailed demographic and clinical data of the three study cohorts are reported in Table 1. Median age (MMN: 60, range 28–92; MADSAM: 59, range 34–75; ALS 65, range 48–78 years) and sex distribution were similar across the study groups. Disease duration was longer in MMN and MADSAM patients compared to ALS patients (MMN vs. ALS *p* < 0.001; MADSAM vs. ALS *p* < 0.01). In MMN and MADSAM, disease onset was most often at the upper limb (MMN: 20/26, 77%; MADSAM: 10/16, 63%) and unilateral (MMN: 24/26, 92%; MADSAM: 13/16, 88%), while in patients with ALS lower limbs (10/18, 55%, bulbar onset excluded) and bilateral (15/22, 68%) affection were typical. The MRC sum score and the upper limb MRC score were higher in patients with MADSAM compared to those with ALS (*p* < 0.01, each). Patients with MMN also showed a trend toward higher MRC scores compared to ALS, although this difference did not reach statistical significance (Table 1). Conditions and risk factors potentially associated with polyneuropathy (PNP) were present in a minority of patients (Supplemental Table 1), and no electrophysiological features of generalized PNP were observed.

**Table 1:** Demographic and clinical data of the study cohort

	MMN (a) (n = 26)	MADSAM (b) (*n* = 16)	ALS (c) (*n* = 22)	p-value (a vs. c; b vs. c)
Sex (M/F)	15/11	12/4	15/7	n.s
Age (years)^a^	60 (28–82)	59 (34–75)	65 (48–78)	n.s
Duration of symptom onset (years)	6.5 (0–24)	5.5 (0–33)	1.0 (0–9)	a vs. c *p* < 0.001 b vs. c *p* < 0.01
Disease onset (bulbar:upper limbs:lower limbs)	0/20/6	0/10/6	4/8/10	Upper limb vs. lower limb a vs. c *p* < 0.001 b vs. c n.s
Disease onset (unilateral:bilateral)	24/2	14/2	7/15	a vs. c p < 0.001 b vs. c p < 0.001
Current therapy				
- IVIG (yes/no)	24/2	14/2	0/22	−
- Time since last IVIG (days)	28 (5–56)	28 (14–93)	N.A	−
- Dosage (g/kg body weight)	1.0 (0.4–1.7)	1.0 (0.4–1.0)	N.A	−
- Riluzol (yes/no)	0/0	0/0	20/2	−
Clinical examination				
- Upper limb affected				
Yes/no	23/3	14/2	18/4	n.s
Unilateral:bilateral	12/10	3/11	1/17^b^	n.s
- Lower limb affected				
Yes/no	13/13	11/5	15/7	n.s
Unilateral:bilateral	4/9	1/10	1/14	n.s
- MRC sum score	113 (42–119)	118 (75–120)	103 (16–120)	b vs. c p < 0.01
Upper limb	58 (17–60)	59 (55–60)	53 (16–60)	b vs. c p < 0.01
Lower limb	60 (25–60)	59 (20–60)	57 (0–60)	n.s
- ODSS score	3 (0–10)	2.5 (0–6)	N.A	−
Upper limb	2 (0–4)	1.5 (0–2)	N.A	−
Lower limb	1.5 (0–6)	1.5 (0–4)	N.A	−
- ALSFRS-R	N.A	N.A	37 (12–47)	−

*ALS*   amyotrophic lateral sclerosis, *ALSFRS-R*   Revised Amyotrophic Lateral Sclerosis Functional Rating Scale, *F*   female, *IVIG*   intravenous immunoglobulin, *M*   male, *MADSAM*   multifocal acquired demyelinating sensory and motor neuropathy, *MMN*   multifocal motor neuropathy, *MRC*   medical research council, *N.A.*   not applicable, *n.s.*   not significant, *ODSS*   overall disability sum score

^a^ data are given as median, range in brackets; ^b^ bulbar excluded

### Clinical signs of SH are equal in patients with MMN, MADSAM, and ALS

**Fig. 1:**
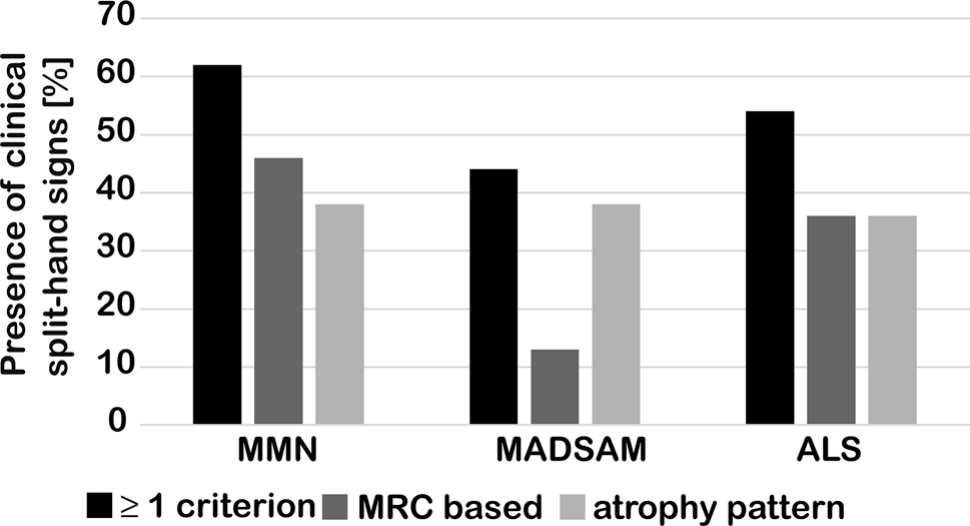
Frequency of clinical split-hand signs. The bar chart illustrates the frequency of clinical split-hand signs in patients with MMN, MADSAM, and ALS. The combined criterion was defined as the presence of either MRC-based weakness and/or a characteristic atrophy pattern. This was observed at similar rates across all three groups. No differences were found between MMN, MADSAM, and ALS, when comparing the individual components. Number of investigated participants: MMN = 26, MADSAM = 16, ALS = 22. *ALS*   amyotrophic lateral sclerosis, *MADSAM*   multifocal acquired demyelinating sensory and motor neuropathy, *MMN*   multifocal motor neuropathy, *MRC*   Medical Research Council

Patients with MMN and MADSAM fulfilled the clinical criteria for split-hand syndrome (MMN: 16/26, 62%; MADSAM; 7/16; 44%) in equal frequencies as patients with ALS (12/22, 54%; *p* > 0.05) (Table 2, Fig. 1). Separate analysis of the individual components, namely, the (i) MRC-based weakness and the (ii) characteristic atrophy pattern, revealed similar frequencies across all patient groups (Table 2, Fig. 1). Likewise, the distribution pattern (unilateral vs. bilateral) did not differ notably between patients with neuropathy and those with ALS (Table 2)**.**

**Table 2:** Clinical split-hand criteria

	MMN (a) (*n* = 26)	MADSAM (b) (*n* = 16)	ALS (c) (*n* = 22)	p-value (a vs. c; b vs. c)
MRC and/or atrophy pattern^a^ (yes/no)	16/10	7/9	12/10	n.s
MRC-based yes/no	12/14	2/14	8/14	n.s
Unilateral:bilateral	11/1	1/1	5/3	n.s
Atrophy pattern				
Yes/no	10/16	6/10	8/14	n.s
Unilateral:bilateral	8/2	3/3	2/6	a vs. c p < 0.05

*ALS*   amyotrophic lateral sclerosis, *MADSAM*   multifocal acquired demyelinating sensory and motor neuropathy, *MMN*   multifocal motor neuropathy, *MRC*   medical research council, *n.s.*   not significant

^a^ The MRC-based and atrophy-based criteria were not fully overlapping; some patients fulfilled one of the two criteria, while others fulfilled both

### Split-hand indices are abnormal in patients with MMN and MADSAM to a similar extent as in ALS

**Fig. 2:**
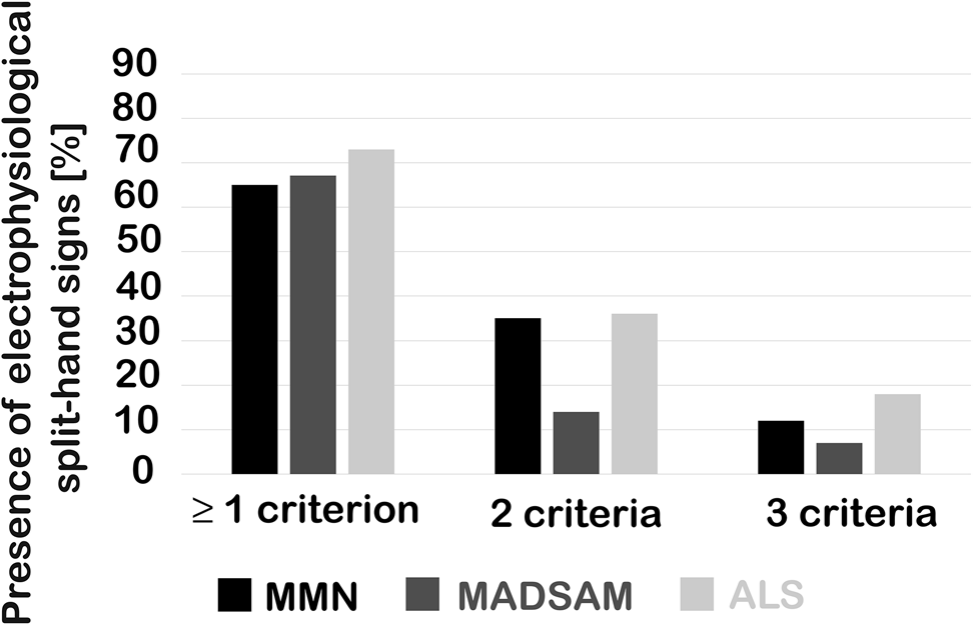
Frequency of electrophysiological split-hand signs**.** The bar chart illustrates the frequency of electrophysiological split-hand signs in patients with MMN, MADSAM, and ALS. The criterion “ ≥ 1” indicates that at least one CMAP ratio and/or the SI fell below the defined cut-off. This was observed at similar frequencies across all three groups. The presence of two or more abnormal electrophysiological criteria was less common but occurred at comparable rates among MMN, MADSAM, and ALS patients. Number of investigated participants: MMN = 26, MADSAM = 16, ALS = 22. *ALS*   amyotrophic lateral sclerosis, *CMAP*   compound muscle action potential, *MADSAM*   multifocal acquired demyelinating sensory and motor neuropathy, *MMN*   multifocal motor neuropathy, *SI*   split-hand index

**Fig. 3:**
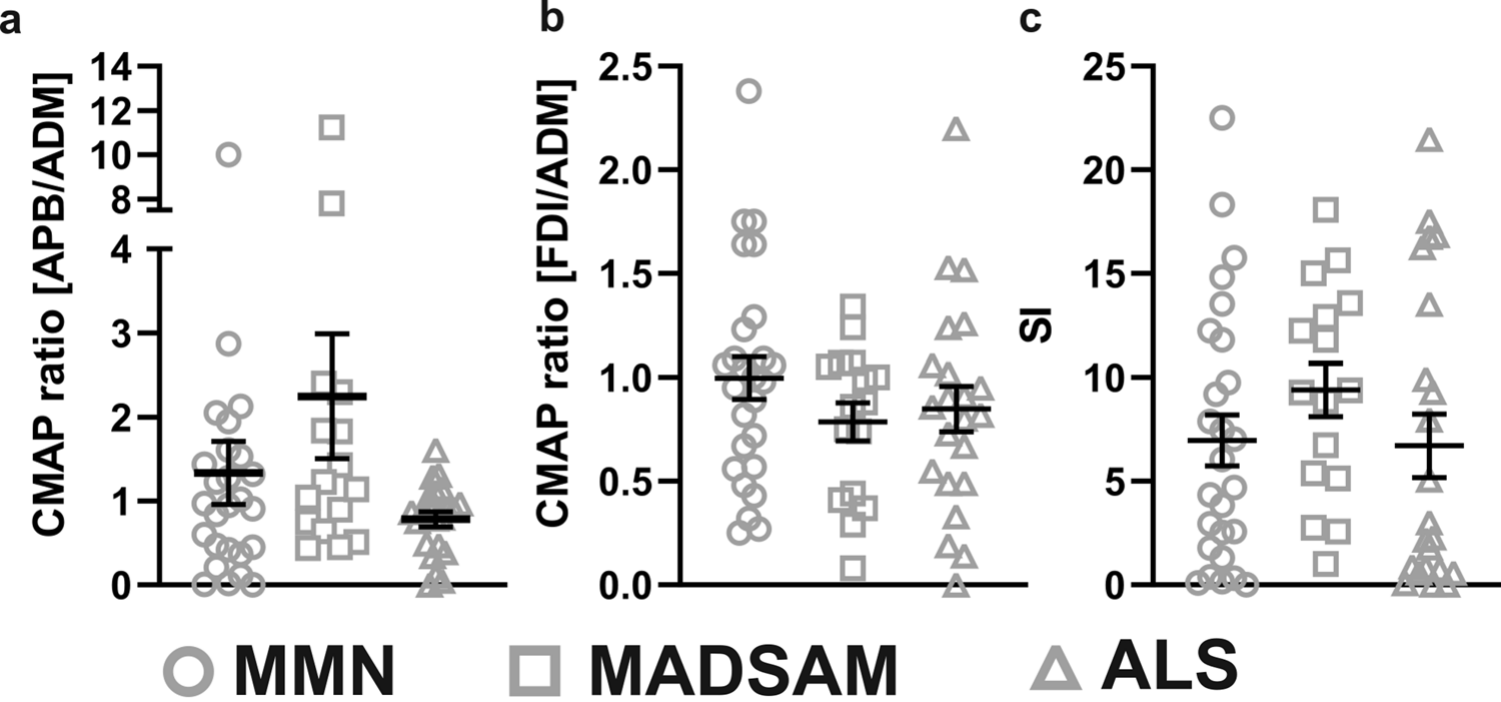
Comparison of the CMAP ratios and the SI. The scatter blots show individual values for three electrophysiological split-hand markers: the CMAP ratio of APB to ADM (**a**), the CMAP ratio of FDI to ADM (**b**), and the SI (**c**). No differences were observed between MMN, MADSAM, and ALS. Patients falling below the predefined electrophysiological split-hand thresholds (APB/ADM ratio < 0.6, FDI/ADM ratio < 0.9, and SI < 5.2) were MMN (a 10/26, b 12/26, c 15/26), MADSAM (a 4/16, b 10/16, c 5/16), and ALS (a 9/22, b 15/22, c 13/22). Categorical comparison of positivity rates showed no group differences. Number of investigated participants: MMN = 26, MADSAM = 16, ALS = 22. *ADM*  abductor digiti minimi, *ALS*   amyotrophic lateral sclerosis, *APB*   abductor pollicis brevis, *CMAP*   compound muscle action potential, *FDI*   first dorsal interosseous, *MADSAM*   multifocal acquired demyelinating sensory and motor neuropathy, *MMN*   multifocal motor neuropathy, *SI*   split-hand index

17/26 (65%) MMN and 11/16 (69%) MADSAM patients fulfilled at least one of the electrophysiological split-hand criteria, comparable to the frequency observed in patients with ALS (16/22, 73%; *p* > 0.05) (Fig. 2). The distribution of patients meeting more than one split-hand criterion did not differ between the neuropathy and ALS groups (see Fig. 2 for details). Similarly, CMAP ratios, the SI, and the positivity rates of the individual electrophysiological split-hand criteria showed no differences between MMN, MADSAM, and ALS (all *p* > 0.05) (Fig. 3, Table 3). In addition, categorical comparisons of positivity rates below the predefined diagnostic thresholds (APB/ADM < 0.6, FDI/ADM < 0.9, SI < 5.2) revealed no group differences (all *p* > 0.05) (Fig. 3).

**Table 3:** Electrophysiological split-hand criteria

	MMN (a) (*n* = 26)	MADSAM (b) (*n* = 16)	ALS (c) (*n* = 22)	p-value (a vs. c; b vs. c)
CMAP amplitude (APB/ADM) < 0.6 (yes/no)	10/16	4/12	9/13	n.s
CMAP amplitude (FDI/ADM) < 0.9 (yes/no)	12/14	10/6	15/7	n.s
SI < 5.2 (yes/no)	15/11	5/11	13/9	n.s

*ADM*   abductor digiti minimi, *ALS*   amyotrophic lateral sclerosis, *APB*   abductor pollicis brevis, *CMAP*   compound muscle action potential, *FDI*   first dorsal interosseous, *MADSAM*   multifocal acquired demyelinating sensory and motor neuropathy, *MMN*   multifocal motor neuropathy, *SI*   split-hand index, *n.s.*   not significant

### Split-hand syndrome is unrelated to disease stage or severity

**Fig. 4:**
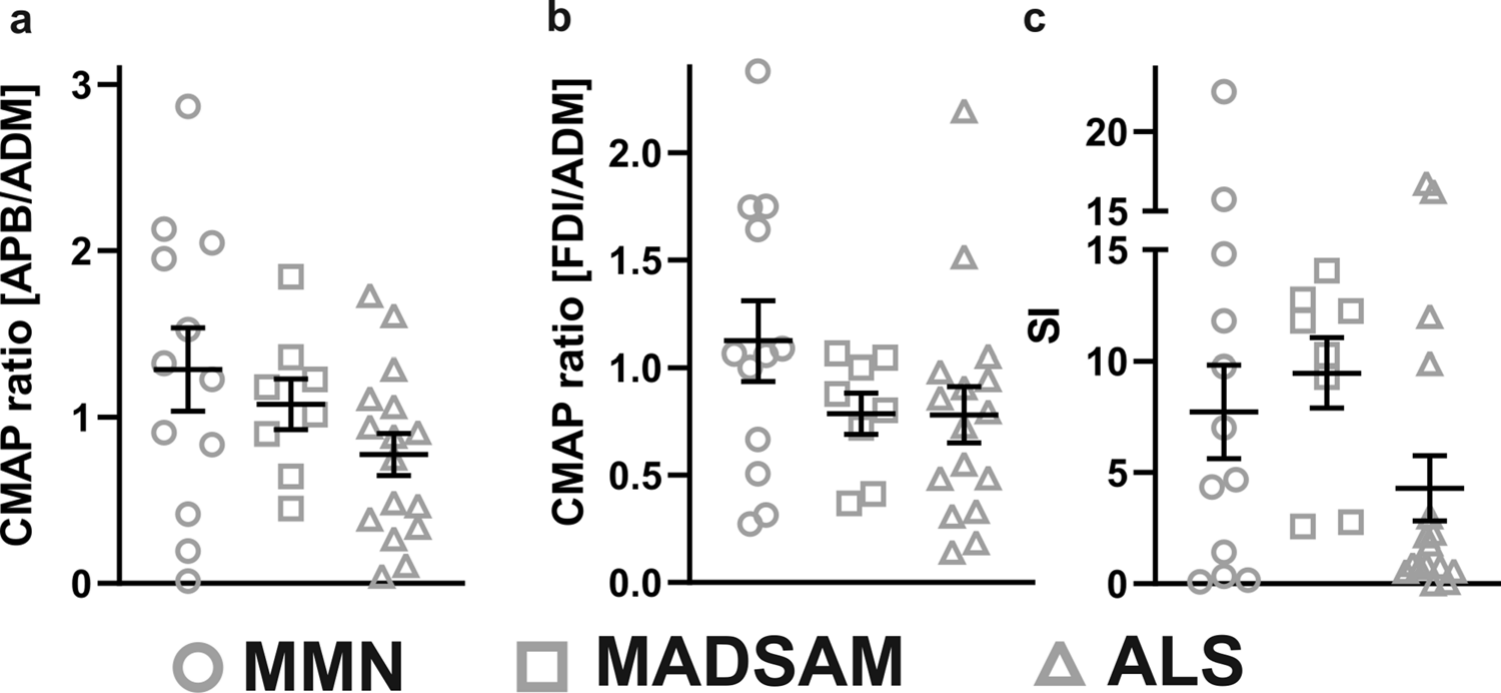
Comparison of the CMAP ratios and the SI in the subgroup patients with shorter disease duration. The scatter blots show individual values for three electrophysiological split-hand markers: the CMAP ratio of APB to ADM (**a**), the CMAP ratio of FDI to ADM (**b**), and the SI (**c**). Those parameters were compared between MMN or MADSAM patients with shorter disease duration and those with ALS. No differences were observed between those neuropathy patients and patients with ALS. Patients falling below the predefined electrophysiological split-hand thresholds (APB/ADM ratio < 0.6, FDI/ADM ratio < 0.9, and SI < 5.2) were MMN (a 3/12, b 4/12, c 6/12), MADSAM (a 1/8, b 4/8, c 2/8), and ALS (a 7/22, b 9/22, c 17/22). Categorical comparison of positivity rates showed a lower frequency of SI positivity in MADSAM compared to ALS in this subgroup. Number of investigated participants: MMN = 12, MADSAM = 8, ALS = 22. *ADM*   abductor digiti minimi, *ALS*   amyotrophic lateral sclerosis, *APB*   abductor pollicis brevis, *CMAP*   compound muscle action potential, *FDI*   first dorsal interosseous, *MADSAM*   multifocal acquired demyelinating sensory and motor neuropathy, *MMN*   multifocal motor neuropathy, *SI*   split-hand index

Due to observed differences in disease severity and duration between neuropathy and ALS patients, we examined whether these factors influence the occurrence of the split-hand phenomenon. Correlation analyses were conducted between disease severity (assessed by upper limb MRC scores and ODSS in neuropathy patients, and ALSFRS-R in ALS patients) and CMAP ratios for split-hand syndromes. We also analyzed whether disease duration was associated with the presence of electrophysiological split-hand syndrome. No correlations were found between those parameters neither in patients with neuropathy nor in those with ALS (Supplemental Table 2). To assess whether the split-hand phenomenon is already detectable in shorter disease duration MMN or MADSAM, at the stage when differentiation from ALS is more critical, we used the median disease duration of the MMN group (6.5 years) as a threshold to define a subgroup of MMN and MADSAM patients with shorter disease duration for further analysis. Although this approach allowed comparison with ALS patients at relatively earlier stages, it does not fully represent the clinically most relevant early diagnostic window of the first 1–2 years. Electrophysiological split-hand criteria in this shorter disease duration subgroup were present at comparable overall frequencies to those observed in patients with ALS (Fig. 4). In addition, categorical comparisons of criterion-level positivity rates revealed no group differences, except for the SI in the subgroup with shorter disease duration, which was less frequently positive in MADSAM than in ALS (Fig. 4); continuous SI values did not differ between groups.

### Retrospective subset analysis confirms equal split-hand frequency across newly diagnosed MMN/MADSAM and ALS

**Fig. 5:**
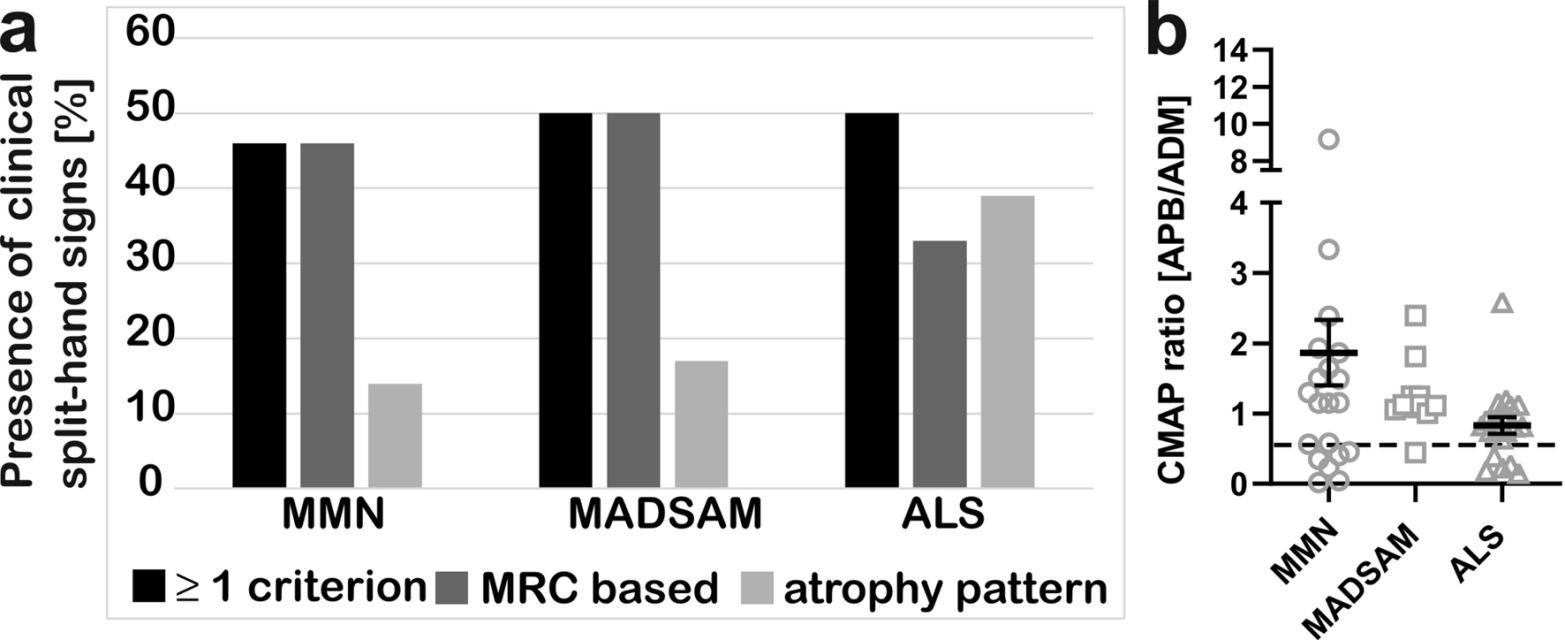
Split-hand occurrence in retrospective dataset. The bar chart (**a**) illustrates the frequency of clinical split-hand signs in the retrospective dataset comprising patients with MMN, MADSAM, and ALS. The combined criterion was defined as the presence of either MRC-based weakness and/or a characteristic atrophy pattern and occurred at similar rates across all three groups. No differences were observed between MMN, MADSAM, and ALS, when comparing the individual components. The scatter blots (**b**) show individual values for the CMAP ratio of APB to ADM with no difference between groups. Percentages of patients falling below the threshold were 32% in MMN, 8% in MADSAM, and 33% in ALS. Number of investigated participants: MMN = 22, MADSAM = 12, ALS = 18. *ADM*   abductor digiti minimi, *ALS*   amyotrophic lateral sclerosis, *APB*   abductor pollicis brevis, *CMAP*   compound muscle action potential, *MADSAM*   multifocal acquired demyelinating sensory and motor neuropathy, *MMN*   multifocal motor neuropathy, *MRC*   Medical Research Council

To assess whether the split-hand syndrome is already present at initial clinical presentation, we analyzed a retrospective subset of newly diagnosed, treatment-naïve patients with the aim of documenting early split-hand features rather than evaluating the diagnostic performance of specific electrophysiological indices. Clinical split-hand signs occurred at similar frequencies in MMN (10/22, 45%), MADSAM (6/12, 50%), and ALS (9/18, 50%). The individual clinical components (MRC-based weakness and characteristic atrophy pattern) also showed comparable distributions across all groups (Fig. 5a). As FDI recordings were not obtained in routine electrophysiological assessments at first presentation, evaluation of the electrophysiological split-hand was restricted to the CMAP amplitude APB/ADM ratio, which precluded calculation of the SI in this retrospective cohort. This parameter was met at similar frequencies in MMN, MADSAM, and ALS (Fig. 5b).

### Split-hand criteria lack diagnostic discriminatory power between MMN and ALS

Predictive modeling revealed that neither the clinical split-hand criterion (based on muscle weakness and/or atrophy), nor the electrophysiological findings offered sufficient discriminatory power to distinguish MMN from ALS (Supplemental Table 3). Also, the combination of these binary parameters yielded an AUC of 0.58, with a sensitivity of 86.4% and a specificity of 34.6%, indicating limited clinical utility. Analysis of the retrospective cohort confirmed this pattern, demonstrating similarly low discriminatory performance across all split-hand criteria (Supplemental Table 3). Even the atrophy-based criterion, which yielded the highest specificity (85.7%), failed to meaningful improve overall diagnostic accuracy.

ROC curve analyses of continuous electrophysiological parameters showed no relevant diagnostic separation (Supplemental Table 4). While combining these variables slightly increased the AUC to 0.61 (sensitivity: 86.4%; specificity: 34.6%), overall discriminative performance remained weak.

## Discussion

Our study provides the first comprehensive clinical and electrophysiological assessment of split-hand syndrome in patients with MMN and MADSAM and the first direct comparison with ALS patients. We demonstrate that the clinical and electrophysiological split-hand syndrome occurred at comparable frequencies in MMN, MADSAM, and ALS. Analyses of frequencies, distribution patterns, and individual electrophysiological parameters did not reveal features that reliably distinguish those immune-mediated, treatable neuropathies from ALS. A fixed SI cut-off of 5.2 was applied in accordance with previous studies [7]. Although age-related effects on split-hand parameters have been reported [9], age-adjusted normative values are not yet uniformly established and fixed thresholds remain widely used in the literature [1, 7, 10]. These findings underscore that this phenomenon is not unique to ALS and underline the need for more precise diagnostic criteria [11, 14, 16].

Consistent with the known clinical profiles of MMN and MADSAM [20, 34], most neuropathy patients in our cohort presented with unilateral upper limb onset. In contrast, ALS typically manifests with spinal onset in about two-thirds of patients, with distal or proximal weakness in either upper or lower-limbs and an almost balanced distribution [35, 36]. Against this background, our cohorts represent archetypical MMN/MADSAM and lower motor neuron (LMN)-dominant ALS patients. Interestingly, despite the generally more benign course of MMN and MADSAM [20, 34, 37], our patients showed lower MRC sum scores than the ALS patients. This likely reflects the markedly longer disease duration in our MMN and MADSAM patients, whereas ALS patients were assessed soon after diagnosis. The ALS cohort, however, represents the early disease stage in which diagnostic uncertainty is greatest, and misclassification of treatable neuropathies as ALS may delay immunotherapy and worsen prognosis.

An influence of disease duration and severity on the split-hand results appears unlikely, as no correlation with split-hand indices was found in MMN, MADSAM, or ALS. The presence of the split-hand phenomenon even in MMN and MADSAM patients with short disease duration further illustrates its limited diagnostic value, particularly since early stages would be the critical window for a useful marker. Although categorical analysis revealed a lower frequency of SI positivity in MADSAM compared to ALS in patients with shorter disease duration, this finding was confined to a small disease-duration-defined subgroup, was not mirrored by other split-hand indices, and did not translate into differences in continuous SI values or overall diagnostic performance. Notably, our retrospective cohort of treatment-naïve patients showed the same distribution of split-hand signs across MMN, MADSAM, and ALS, indicating that the phenomenon is present in immune-mediated neuropathies from the earliest disease stage and therefore offers no diagnostic advantage during initial evaluation. Importantly, some patients had conditions and risk factors associated with PNP; however, no electrophysiological pattern consistent with generalized PNP was observed, making a relevant confounding effect on CMAP-based split-hand parameters unlikely.

Although frequencies and distribution patterns were similar across groups, our findings argue against a purely cortical origin of the split-hand phenomenon. The presence of the split-hand features in immune-mediated peripheral neuropathies in our cohort indicates that cortical mechanisms alone cannot account for this pattern. While a cortical origin has been proposed in ALS, increasing evidence supports additional peripheral mechanisms, as reflected by the occurrence of split-hand in various motor and hereditary neuropathies [10, 11, 16]. The recent study by Bertini et al. in CMTX1 [16] further broadens the spectrum of disorders associated with split-hand syndrome. Together with our findings in MMN and MADSAM, these data indicate that split-hand features may occur across a range of non-fatal peripheral neuropathies and therefore have limited specificity for ALS. This highlights the importance of interpreting split-hand signs within the broader clinical and electrophysiological context rather than considering them a disease-specific marker. A peripheral contribution may involve structural and functional alterations of motor axons or neuromuscular junctions, rendering thenar-innervating motor units more susceptible to conduction failure than hypothenar units [10, 16].

In MMN, motor excitability studies indicate marked abnormalities best explained by increased Barrett–Barrett conductance, indicating demyelination or disruption of the paranodal seal with sparing of sensory fibers [38, 39]. This paranodal/myelin dysfunction diminishes the conduction reserve selectively in motor axons and, combined with intrinsic physiological differences between APB/FDI and ADM motor units, can reproduce the split-hand pattern. In MADSAM, immune-mediated disruption of paranodal protein complexes causes conduction block and similar current leakage, likely affecting the same selectively vulnerable motor units [40, 41]. Our findings of thenar–hypothenar dissociation in both MMN and MADSAM therefore reinforce the key role of the peripheral nervous system in the genesis of the split-hand sign and underscore that this phenomenon is not unique to ALS, a conclusion further supported by its low discriminative value in ROC curve analyses of clinical signs.

Our study has several limitations. The sample size, particularly of the ALS cohort, was relatively small. However, MMN and MADSAM are rare disorders, and recruitment was limited to patients undergoing standardized clinical and neurophysiological assessment at a single tertiary referral center. Therefore, our findings should be interpreted as exploratory rather than definitive estimates of diagnostic specificity. The present study focused on established clinical and conventional neurophysiological split-hand measures. It cannot be excluded that more sensitive quantitative techniques, such as motor unit number estimation/index, axonal excitability studies, MRI-based muscle volumetry, or quantitative hand muscle measurements, may provide greater discriminatory value between ALS and immune-mediated motor neuropathies. In addition, the relatively small sample size may have limited our ability to detect moderate group differences that could potentially be identified using such approaches. Although patients were prospectively recruited, some information, such as disease onset and site of onset, had to be obtained retrospectively from medical records. As most patients were first diagnosed in our outpatient clinic, records were generally available, but recall and documentation bias cannot be fully excluded. Moreover, several MMN and MADSAM patients were receiving treatment with different doses and intervals. To reduce this confounder, examinations were consistently performed immediately before the next scheduled infusion, ensuring a standardized assessment at the end of the individual treatment cycle. In addition, diagnostic accuracy analyses (ROC curves, sensitivity, and specificity) were restricted to comparisons between ALS and MMN, reflecting the clinically most relevant differential diagnosis. Formal ROC analyses including MADSAM were not performed because the relatively small MADSAM cohort precluded robust multivariable diagnostic modeling. However, MADSAM was included in all group-level and criterion-level analyses; these findings were not consistent across split-hand measures and did not support meaningful diagnostic discrimination from ALS. Furthermore, the evaluation of the split-hand pattern partly relied on visual inspection of muscle atrophy, which is inherently subjective and may not always correspond to MRC-based strength grading, particularly in the presence of mild or compensated weakness. Although assessments were restricted to two experienced examiners, formal inter-rater reliability was not evaluated and therefore observer-related variability cannot be excluded.

Despite these limitations, this is the first study to directly compare the split-hand phenomenon in MMN, MADSAM, and ALS. To mitigate potential imbalances in disease duration and treatment status, we additionally analyzed a retrospective cohort of newly diagnosed, treatment-naïve patients. Although this cohort reflects the stage at which patients first present for clinical evaluation, the broad ranges in symptom duration indicate that not all patients were within the earliest diagnostic window. This approach, however, is constrained by the inherent limitations of initial clinical documentation, particularly the uncertainty as to whether all relevant hand muscles were systematically assessed when no weakness or atrophy was noted. Overall, our results confirm that split-hand is not a specific or consistently discriminatory biomarker for ALS, despite isolated differences observed in subgroup analyses, and demonstrate its presence in immune-mediated neuropathies. These findings emphasize the need for large-scale, longitudinal studies integrating clinical, neurophysiological, and molecular approaches to establish reliable tools for differential diagnosis and improved clinical decision-making in motor neuropathies.

In summary, split-hand features were observed in MMN, MADSAM, and ALS at comparable frequencies. Analyses of individual electrophysiological indices, cut-off-based positivity rates, and continuous parameters revealed no consistent differences that would support reliable diagnostic discrimination between these disease groups. Although isolated differences emerged in subgroup analyses, these findings were not replicated across other indices and did not translate into meaningful diagnostic performance and should therefore be interpreted with caution given the small sample size of the subgroup. Taken together, our results indicate that split-hand syndrome is not specific to ALS and has limited value as a standalone marker for distinguishing ALS from immune-mediated motor neuropathies. Larger longitudinal studies integrating clinical, neurophysiological, and molecular biomarkers will be required to improve early differential diagnosis.

## Supplementary Information

Below is the link to the electronic supplementary material.

Supplementary file1 (DOCX 17 KB)

## Data Availability

All data generated or analyzed during this study are included in this published article.

## References

[1] Lu WZ, Lin HA, Hou SK, Lee CF, Bai CH, Lin SF (2022) Split-hand index for amyotrophic lateral sclerosis diagnosis: a frequentist and Bayesian meta-analysis. Clin Neurophysiol 143:56–66. 10.1016/j.clinph.2022.08.020 36116424 10.1016/j.clinph.2022.08.020

[2] Hu N, Wang J, Liu M (2021) Split hand in amyotrophic lateral sclerosis: a systematic review and meta-analysis. J Clin Neurosci 90:293–301. 10.1016/j.jocn.2021.06.015 34275566 10.1016/j.jocn.2021.06.015

[3] Wilbourn A, Sweeney P (1994) Dissociated wasting of medial and lateral hand muscles with motor neuron disease. Can J Neurol Sci 21(Suppl 2):S9

[4] Zoccolella S, Giugno A, Logroscino G (2022) Split phenomena in amyotrophic lateral sclerosis: current evidences, pathogenetic hypotheses and diagnostic implications. Front Neurosci 16:1100040. 10.3389/fnins.2022.1100040 36699516 10.3389/fnins.2022.1100040 PMC9868395

[5] Eisen A, Kuwabara S (2012) The split hand syndrome in amyotrophic lateral sclerosis. J Neurol Neurosurg Psychiatry 83(4):399–403. 10.1136/jnnp-2011-301456 22100761 10.1136/jnnp-2011-301456

[6] Kim DG, Hong YH, Shin JY, Park KH, Sohn SY, Lee KW et al (2016) Split-hand phenomenon in amyotrophic lateral sclerosis: A motor unit number index study. Muscle Nerve 53(6):885–888. 10.1002/mus.24958 26509758 10.1002/mus.24958

[7] Menon P, Kiernan MC, Yiannikas C, Stroud J, Vucic S (2013) Split-hand index for the diagnosis of amyotrophic lateral sclerosis. Clin Neurophysiol 124(2):410–416. 10.1016/j.clinph.2012.07.025 23017503 10.1016/j.clinph.2012.07.025

[8] Wilbourn AJ (2000) The “split hand syndrome.” Muscle Nerve 23(1):138. 10.1002/(sici)1097-4598(200001)23:1<138::aid-mus22>3.0.co;2-7 10590421 10.1002/(sici)1097-4598(200001)23:1<138::aid-mus22>3.0.co;2-7

[9] Zoccolella S, Milella G, Giugno A, Devitofrancesco V, Damato R, Tamburrino L et al (2024) Neurophysiological indices for split phenomena: correlation with age and sex and potential implications in amyotrophic lateral sclerosis. Front Neurol 15:1371953. 10.3389/fneur.2024.1371953 38515451 10.3389/fneur.2024.1371953 PMC10956616

[10] Corcia P, Bede P, Pradat PF, Couratier P, Vucic S, de Carvalho M (2021) Split-hand and split-limb phenomena in amyotrophic lateral sclerosis: pathophysiology, electrophysiology and clinical manifestations. J Neurol Neurosurg Psychiatry 92(10):1126–1130. 10.1136/jnnp-2021-326266 34285065 10.1136/jnnp-2021-326266

[11] Shibuya K, Misawa S, Uzawa A, Sawai S, Tsuneyama A, Suzuki YI et al (2020) Split hand and motor axonal hyperexcitability in spinal and bulbar muscular atrophy. J Neurol Neurosurg Psychiatry 91(11):1189–1194. 10.1136/jnnp-2020-324026 32934003 10.1136/jnnp-2020-324026

[12] Kalita J, Kumar S, Misra UK, Neyaz Z (2017) Split hand index and ulnar to median ratio in Hirayama disease and amyotrophic lateral sclerosis. Amyotroph Lateral Scler Frontotemporal Degener 18(7–8):598–603. 10.1080/21678421.2017.1336561 28616933 10.1080/21678421.2017.1336561

[13] Vucic S (2020) Split-hand sign: clinical feature of spinal bulbar muscular atrophy? J Neurol Neurosurg Psychiatry 91(11):1143–1144. 10.1136/jnnp-2020-324969 33004430 10.1136/jnnp-2020-324969

[14] Fang J, Liu MS, Guan YZ, Du H, Li BH, Cui B et al (2016) Pattern differences of small hand muscle atrophy in amyotrophic lateral sclerosis and mimic disorders. Chin Med J (Engl) 129(7):792–798. 10.4103/0366-6999.178953 26996473 10.4103/0366-6999.178953 PMC4819298

[15] Singh RJ, Preethish-Kumar V, Polavarapu K, Vengalil S, Prasad C, Nalini A (2017) Reverse split hand syndrome: dissociated intrinsic hand muscle atrophy pattern in Hirayama disease/brachial monomelic amyotrophy. Amyotroph Lateral Scler Frontotemporal Degener 18(1–2):10–16. 10.1080/21678421.2016.1223140 27575868 10.1080/21678421.2016.1223140

[16] Bertini A, Moscatelli M, Ciano C, Verri M, Cavalca E, Sconfienza LM et al (2025) Split hand syndrome in Charcot-Marie-Tooth Disease Type X1 (CMTX1): a clinical, neurophysiological, and radiological study. Eur J Neurol 32(5):e70188. 10.1111/ene.70188 40345990 10.1111/ene.70188 PMC12062869

[17] Van den Bergh PYK, van Doorn PA, Hadden RDM, Avau B, Vankrunkelsven P, Allen JA et al (2021) European Academy of Neurology/Peripheral Nerve Society guideline on diagnosis and treatment of chronic inflammatory demyelinating polyradiculoneuropathy: report of a joint Task Force-Second revision. Eur J Neurol 28(11):3556–3583. 10.1111/ene.14959 34327760 10.1111/ene.14959

[18] Shelly S, Mills JR, Martinez-Thompson JM, Rofforth MM, Pittock SJ, Mandrekar J et al (2020) IgM-gammopathy strongly favours immune treatable MMN and MADSAM over ALS. J Neurol Neurosurg Psychiatry 91(3):324–326. 10.1136/jnnp-2019-321977 31757814 10.1136/jnnp-2019-321977

[19] Rizea RE, Corlatescu AD, Costin HP, Dumitru A, Ciurea AV (2024) Understanding amyotrophic lateral sclerosis: pathophysiology, diagnosis, and therapeutic advances. Int J Mol Sci. 10.3390/ijms25189966 39337454 10.3390/ijms25189966 PMC11432652

[20] Claytor B, Polston D, Li Y (2025) Multifocal motor neuropathy: a narrative review. Muscle Nerve 71(4):512–534. 10.1002/mus.28349 39936246 10.1002/mus.28349 PMC11887531

[21] Stino AM, Barohn RJ, Katz JS, Dimachkie MM (2025) Chronic inflammatory demyelinating polyneuropathy and variants. Neurol Clin 43(4):761–780. 10.1016/j.ncl.2025.05.015 41161995 10.1016/j.ncl.2025.05.015

[22] Allen JA, Clarke AE, Harbo T (2024) A practical guide to identify patients with multifocal motor neuropathy, a treatable immune-mediated neuropathy. Mayo Clin Proc Innov Qual Outcomes 8(1):74–81. 10.1016/j.mayocpiqo.2023.12.002 38283096 10.1016/j.mayocpiqo.2023.12.002 PMC10819864

[23] Yeh WZ, Dyck PJ, van den Berg LH, Kiernan MC, Taylor BV (2020) Multifocal motor neuropathy: controversies and priorities. J Neurol Neurosurg Psychiatry 91(2):140–148. 10.1136/jnnp-2019-321532 31511307 10.1136/jnnp-2019-321532

[24] Kronlage M, Knop KC, Schwarz D, Godel T, Heiland S, Bendszus M et al (2019) Amyotrophic lateral sclerosis versus multifocal motor neuropathy: utility of MR neurography. Radiology 292(1):149–156. 10.1148/radiol.2019182538 31063079 10.1148/radiol.2019182538

[25] Loewenbrück KF, Werner R, Günther R, Dittrich M, Klingenberger R, Reichmann H et al (2021) One nerve suffices: a clinically guided nerve ultrasound protocol for the differentiation of multifocal motor neuropathy (MMN) and amyotrophic lateral sclerosis (ALS). J Neurol 268(4):1495–1507. 10.1007/s00415-020-10323-6 33355881 10.1007/s00415-020-10323-6 PMC7990818

[26] Foesleitner O, Knop KC, Lindenau M, Preisner F, Bäumer P, Heiland S et al (2023) Quantitative MR neurography in multifocal motor neuropathy and amyotrophic lateral sclerosis. Diagnostics (Basel). 10.3390/diagnostics13071237 37046455 10.3390/diagnostics13071237 PMC10093201

[27] Cats EA, Jacobs BC, Yuki N, Tio-Gillen AP, Piepers S, Franssen H et al (2010) Multifocal motor neuropathy: association of anti-GM1 IgM antibodies with clinical features. Neurology 75(22):1961–1967. 10.1212/WNL.0b013e3181ff94c2 20962291 10.1212/WNL.0b013e3181ff94c2

[28] Kleinveld VEA, Keritam O, Horlings CGC, Cetin H, Wanschitz J, Hotter A et al (2024) Multifocal motor neuropathy as a mimic of amyotrophic lateral sclerosis: serum neurofilament light chain as a reliable diagnostic biomarker. Muscle Nerve 69(4):422–427. 10.1002/mus.28054 38334356 10.1002/mus.28054

[29] EFNS/PNS. European Federation of Neurological Societies/Peripheral Nerve Society guideline on management of multifocal motor neuropathy. Report of a joint task force of the European Federation of Neurological Societies and the Peripheral Nerve Society--first revision. J Peripher Nerv Syst. 2010;15(4):295–301. 10.1111/j.1529-8027.2010.00290.x 10.1111/j.1529-8027.2010.00290.x 21199100

[30] Shefner JM, Al-Chalabi A, Baker MR, Cui LY, de Carvalho M, Eisen A et al (2020) A proposal for new diagnostic criteria for ALS. Clin Neurophysiol 131(8):1975–1978. 10.1016/j.clinph.2020.04.005 32387049 10.1016/j.clinph.2020.04.005

[31] Merkies IS, Schmitz PI, van der Meché FG, Samijn JP, van Doorn PA (2002) Clinimetric evaluation of a new overall disability scale in immune mediated polyneuropathies. J Neurol Neurosurg Psychiatry 72(5):596–601. 10.1136/jnnp.72.5.596 11971045 10.1136/jnnp.72.5.596 PMC1737884

[32] Cedarbaum JM, Stambler N, Malta E, Fuller C, Hilt D, Thurmond B et al (1999) The ALSFRS-R: a revised ALS functional rating scale that incorporates assessments of respiratory function BDNF ALS Study Group (Phase III). J Neurol Sci 169(1–2):13–21. 10.1016/s0022-510x(99)00210-5 10540002 10.1016/s0022-510x(99)00210-5

[33] Kimura J (2001) Electrodiagnosis in diseases of nerve and muscle: principles and practice, 3rd edn. Oxford University Press, Oxford

[34] Kuwabara S, Misawa S (2019) Chronic Inflammatory Demyelinating Polyneuropathy. Adv Exp Med Biol 1190:333–343. 10.1007/978-981-32-9636-7_21 31760654 10.1007/978-981-32-9636-7_21

[35] Lynch K (2023) Pathogenesis and presentation of ALS: examining reasons for delayed diagnosis and identifying opportunities for improvement. Am J Manag Care 29(7):S104–S111. 10.37765/ajmc.2023.89390 37433091 10.37765/ajmc.2023.89390

[36] Witzel S, Mayer K, Oeckl P (2022) Biomarkers for amyotrophic lateral sclerosis. Curr Opin Neurol 35(5):699–704. 10.1097/wco.0000000000001094 35942674 10.1097/WCO.0000000000001094

[37] van den Bos MAJ, Geevasinga N, Higashihara M, Menon P, Vucic S (2019) Pathophysiology and diagnosis of ALS: insights from advances in neurophysiological techniques. Int J Mol Sci. 10.3390/ijms20112818 31185581 10.3390/ijms20112818 PMC6600525

[38] Krarup C, Wolfram N, Frahm-Falkenberg S, Graffe CC, Dysgaard T, Al-Zuhairy A et al (2025) Spontaneous muscle activity in multifocal motor neuropathy - insights from axonal excitability testing. Clin Neurophysiol 173:229–238. 10.1016/j.clinph.2024.12.019 39855993 10.1016/j.clinph.2024.12.019

[39] Kovalchuk MO, Franssen H, van den Berg LH, van Schelven LJ, Sleutjes B (2020) Excitability of motor and sensory axons in multifocal motor neuropathy. Clin Neurophysiol 131(11):2641–2650. 10.1016/j.clinph.2020.08.004 32947198 10.1016/j.clinph.2020.08.004

[40] Stephanova DI, Daskalova M (2005) Differences in potentials and excitability properties in simulated cases of demyelinating neuropathies. Part II. Paranodal demyelination. Clin Neurophysiol 116(5):1159–1166. 10.1016/j.clinph.2005.01.005 15826857 10.1016/j.clinph.2005.01.005

[41] Garg N, Park SB, Howells J, Vucic S, Yiannikas C, Mathey EK et al (2019) Conduction block in immune-mediated neuropathy: paranodopathy versus axonopathy. Eur J Neurol 26(8):1121–1129. 10.1111/ene.13953 30882969 10.1111/ene.13953

