# Supplementary material for: Beyond ALS: split-hand syndrome in immune-mediated motor neuropathies
Source: J Neurol. 2026 Jul 17;273(8):471. doi: 10.1007/s00415-026-14006-6 (PMC13379438; doi:10.1007/s00415-026-14006-6)
Supplement: Supplementary file 1 — Supplementary file1 (DOCX 17 KB) [file 415_2026_14006_MOESM1_ESM.docx]

**Supplementary Table 1** Conditions and risk factors potentially associated with polyneuropathy.

| **Comorbidity** | **MMN** (n=26) | **MADSAM** (n=16) | **ALS** (n=22) |
| --- | --- | --- | --- |
| Diabetes mellitus | 1 | 1 | 2 |
| Mild chronic renal insufficiency | 0 | 1 | 2 |
| Thyroid dysfunction | 0 | 1 | 0 |
| Autoimmune disease | 1 | 0 | 0 |
| Regular alcohol consumption (~1 beer/day) | 1 | 0 | 5 |

Relevant conditions were regularly monitored and, where applicable, adequately medically controlled. Abbreviations: ALS = amyotrophic lateral sclerosis, MADSAM = multifocal acquired demyelinating sensory and motor neuropathy, MMN = multifocal motor neuropathy.

**Supplementary Table 2** Correlation analysis between disease duration and severity with the electrophysiological split-hand criteria.

| **Group** | **Clinical criteria** | **CMAP (APB/ADM)** | **CMAP (FDI/ADM)** | **SI** |
| --- | --- | --- | --- | --- |
| MMN (n=26) | MRC score upper limb  Disease duration  Disease severity | r = 0.14, n.s.  r = 0.06, n.s.  r = - 0.21, n.s. | r = - 0.28, n.s.  r = - 0.26, n.s.  r = 0.35, n.s. | r = 0.12, n.s.  r = - 0.35, n.s.  r = - 0.26, n.s. |
| MADSAM (n=16) | MRC score upper limb  Disease duration  Disease severity | r = - 0.27, n.s.  r = 0.26, n.s.  r = 0.24, n.s. | r = - 0.21, n.s.  r = - 0.15, n.s.  r = - 0.33, n.s. | r = - 0.20, n.s.  r = - 0.03, n.s.  r = - 0.61, n.s. |
| ALS (n=22) | MRC score upper limb  Disease duration  Disease severity | r = 0.02, n.s.  r = - 0.24, n.s.  r = 0.13, n.s. | r = - 0.42, n.s.  r = 0.24, n.s.  r = - 0.07, n.s. | r = - 0.13, n.s.  r = 0.15, n.s.  r = - 0.01, n.s. |

Abbreviations: ADM = abductor digiti minimi, APB = abductor pollicis brevis, ALS = amyotrophic lateral sclerosis, CMAP = compound muscle action potential, FDI = first dorsal interosseous, MADSAM = multifocal acquired demyelinating sensory and motor neuropathy, MMN = multifocal motor neuropathy, SI = split-hand index.

**Supplemental Table 3** Diagnostic performance of three split-hand-related criteria in differentiating ALS from MMN.

*In prospective dataset*

| **Parameter** | **Sensitivity (%)** | **Specificity (%)** | **PPV (%)** | **NPV (%)** | **Accuracy (%)** | **AUC** |
| --- | --- | --- | --- | --- | --- | --- |
| Clinical split-hand criterion (MRC-based) | 28.6 | 53.8 | 33.3 | 48.3 | 42.6 | - |
| Atrophy pattern | 38.1 | 61.5 | 44.4 | 55.2 | 51.1 | - |
| Electrophysiological criterion^a^   - CMAP Amplitude   (APB/ADM) < 0.6   - CMAP Amplitude (FDI/ADM) < 0.9 - SI < 5.2 | 71.4  29.0  68.3  63.6 | 34.6  53.8  42.3  38.5 | 46.9  42.9  50.0  46.7 | 60.0  38.9  61.1  55.6 | 51.1  40.4  54.2  50.0 | -  -  -  - |
| Combined binary model^b^ | 86.4 | 34.6 | - | - | - | 0.58 |

*In retrospective dataset*

| **Parameter** | **Sensitivity (%)** | **Specificity (%)** | **PPV (%)** | **NPV (%)** | **Accuracy (%)** | **AUC** |
| --- | --- | --- | --- | --- | --- | --- |
| Clinical split-hand criterion (MRC based) | 33.3 | 54.5 | 37.5 | 50.0 | 45.0 | - |
| Atrophy pattern | 38.9 | 85.7 | 70.0 | 62.1 | 64.1 | - |
| CMAP Amplitude  (APB/ADM) < 0.6 | 33.3 | 68.2 | 46.2 | 55.6 | 52.5 | - |

^a^ at least one out of three fulfilled. ^b^ includes the following parameter: clinical split-hand criterion, atrophy pattern, and electrophysiological criterion.

Abbreviations: ALS = amyotrophic lateral sclerosis, AUC = area under the curve, MMN = multifocal motor neuropathy, NPV = negative predictive value, PPV = positive predictive value.

**Supplemental Table 4** ROC curve analyses of continuous electrophysiological parameters for differentiating ALS from MMN.

|  | **AUC** | **Sensitivity (%)** | **Specificity (%)** |
| --- | --- | --- | --- |
| CMAP (APB/ADM) | 0.41 | 68.2% | 34.6% |
| CMAP (FDI/ADM) | 0.40 | 68.2% | 26.9% |
| SI | 0.47 | 68.2% | 19.2% |
| Combined electrophysiological model | 0.61 | 86.4% | 34.6% |

Abbreviations: ADM = abductor digiti minimi, APB = abductor pollicis brevis, AUC = area under the curve, CMAP = compound muscle action potential, FDI = first dorsal interosseous, SI = split-hand index
